# The role of lactylation in plasma cells and its impact on rheumatoid arthritis pathogenesis: insights from single-cell RNA sequencing and machine learning

**DOI:** 10.3389/fimmu.2024.1453587

**Published:** 2024-10-03

**Authors:** Weicong Fu, Tianbao Wang, Yehong Lu, Tiejun Shi, Qining Yang

**Affiliations:** Department of Orthopedics, Affiliated Jinhua Hospital, Zhejiang University School of Medicine, Jinhua Municipal Central Hospital, Jinhua, Zhejiang, China

**Keywords:** rheumatoid arthritis, lactylation, plasma cells, single-cell RNA sequencing, machine learning

## Abstract

**Introduction:**

Rheumatoid arthritis (RA) is a chronic autoimmune disorder characterized by persistent synovitis, systemic inflammation, and autoantibody production. This study aims to explore the role of lactylation in plasma cells and its impact on RA pathogenesis.

**Methods:**

We utilized single-cell RNA sequencing (scRNA-seq) data and applied bioinformatics and machine learning techniques. A total of 10,163 cells were retained for analysis after quality control. Clustering analysis identified 13 cell clusters, with plasma cells displaying the highest lactylation scores. We performed pathway enrichment analysis to examine metabolic activity, such as oxidative phosphorylation and glycolysis, in highly lactylated plasma cells. Additionally, we employed 134 machine learning algorithms to identify seven core lactylation-promoting genes and constructed a diagnostic model with an average AUC of 0.918.

**Results:**

The RA lactylation score (RAlac_score) was significantly elevated in RA patients and positively correlated with immune cell infiltration and immune checkpoint molecule expression. Differential expression analysis between two plasma cell clusters revealed distinct metabolic and immunological profiles, with cluster 2 demonstrating increased immune activity and extracellular matrix interactions. qRT-PCR validation confirmed that NDUFB3, NGLY1, and SLC25A4 are highly expressed in RA.

**Conclusion:**

This study highlights the critical role of lactylation in plasma cells for RA pathogenesis and identifies potential biomarkers and therapeutic targets, which may offer insights for future therapeutic strategies.

## Introduction

Rheumatoid arthritis (RA) is a chronic autoimmune disorder characterized by inflammation and progressive joint destruction (1, 2). Affecting approximately 0.5-1% of the global population, RA poses a significant burden due to its debilitating nature, reduced quality of life, and increased mortality rates (3–5). Despite extensive research, the precise mechanisms underlying the pathogenesis of RA remain incompletely understood, necessitating ongoing investigations to uncover novel therapeutic targets and biomarkers for early diagnosis and treatment (6).

RA is characterized by persistent synovitis, systemic inflammation, and autoantibody production (7). The disease typically manifests as symmetrical polyarthritis, primarily affecting the small joints of the hands and feet, although large joints and other organs can also be involved (8). The hallmark of RA pathology is the formation of pannus, a hypertrophic synovial tissue that invades and destroys adjacent cartilage and bone (9). This destructive process is driven by a complex interplay of genetic, environmental, and immunological factors.

The immune system plays a central role in the development and progression of RA. A breakdown in immune tolerance leads to the activation of autoreactive T and B cells, which produce autoantibodies such as rheumatoid factor (RF) and anti-citrullinated protein antibodies (ACPAs) (10–12). These autoantibodies form immune complexes that contribute to the activation of complement and the recruitment of inflammatory cells into the synovium (9, 13). T cells, particularly CD4+ T helper cells, are pivotal in RA pathogenesis (14, 15). They interact with antigen-presenting cells and produce a variety of cytokines, including tumor necrosis factor-alpha (TNF-α), interleukin-1 (IL-1), and interleukin-6 (IL-6), which propagate the inflammatory response (16–18). B cells also contribute to RA through autoantibody production, antigen presentation, and cytokine secretion (19, 20).

Recent studies have highlighted the role of metabolic reprogramming in immune cells during RA. Activated immune cells undergo metabolic shifts to meet the increased energy demands and biosynthetic needs required for proliferation, differentiation, and effector functions (21–23). These metabolic alterations include increased glycolysis, oxidative phosphorylation, and lipid metabolism (24–26). Lactylation, a post-translational modification involving the addition of lactate-derived lactyl groups to lysine residues, has emerged as a significant regulatory mechanism in cellular metabolism and gene expression (27). Lactylation can modulate protein function and influence various biological processes, including immune responses and inflammation (28, 29). Given the importance of metabolic reprogramming in RA, understanding the role of lactylation in immune cells, particularly in the context of RA, is of great interest.

Plasma cells, the terminally differentiated form of B cells, are crucial for antibody production (30). In RA, plasma cells are abundant in the synovium and produce autoantibodies that contribute to disease pathogenesis (31, 32). The persistence of plasma cells in the inflamed synovium and their role in sustaining chronic inflammation underscore their importance in RA (33). Recent evidence suggests that plasma cells exhibit distinct metabolic profiles compared to other immune cells (7). The high metabolic activity of plasma cells, characterized by enhanced oxidative phosphorylation and glycolysis, supports their robust antibody production. However, the role of lactylation in plasma cells and its impact on RA progression remain largely unexplored.

Despite advances in understanding RA pathogenesis, current therapeutic strategies remain inadequate, often failing to achieve sustained remission in many patients. The heterogeneity of RA, coupled with its complex molecular and immunological mechanisms, underscores the need for novel biomarkers and therapeutic targets. Recent research has highlighted the importance of lactylation in regulating immune cell function and metabolism, suggesting its potential role in RA pathogenesis. By integrating cutting-edge technologies such as single-cell RNA sequencing (scRNA-seq) and machine learning, we can now analyze large-scale datasets with greater precision. machine learning algorithms, in particular, enable the identification of hidden patterns in gene expression data, the prioritization of key regulatory genes, and the construction of predictive models for disease diagnosis and treatment response. The aim of this study is to investigate the role of lactylation in plasma cells and its impact on RA pathogenesis. By utilizing scRNA-seq data and integrating bioinformatics and machine learning techniques, we seek to identify core lactylation-promoting genes and develop a robust diagnostic model, providing novel insights into RA’s metabolic and immunological mechanisms while highlighting potential biomarkers and therapeutic targets for future intervention.

## Methods

### Data collection

Single-cell RNA sequencing data for RA was collected from the GEO database (https://www.ncbi.nlm.nih.gov/geo/), specifically dataset GSE159117, which includes one RA sample sequenced using the 10X Genomics platform. This dataset was selected because it provides high-resolution insights into the cellular heterogeneity of RA, allowing for the detailed analysis of specific cell types, such as plasma cells, that are crucial for understanding RA pathogenesis. The single-cell data enables us to explore gene expression at an individual cell level, which is essential for investigating the role of lactylation in plasma cells.

In addition, four bulk RNA sequencing datasets were selected from GEO for machine learning purposes to enhance the robustness and generalizability of our findings. These datasets were chosen based on their relevance to RA, their sample sizes, and their inclusion of both RA patients and normal controls, allowing for comprehensive model training and validation. The dataset GSE89408, which includes 28 normal samples and 152 RA samples, was used as the training set due to its large sample size, providing a solid foundation for model development. GSE12021 (9 normal and 12 RA samples), GSE55235 (10 normal and 10 RA samples), and GSE55457 (10 normal and 13 RA samples) were selected as validation sets to ensure that our model could be validated across multiple independent datasets, further strengthening the reliability of the diagnostic model developed in this study. The selection of these datasets was also guided by their representation of both peripheral blood and synovial tissue, offering a broader perspective on the mechanisms of RA across different tissue types.

### qRT-PCR analysis

RNA was extracted from tissues (tumoral and non-tumoral) using an RNA isolation kit (Bioneer, Korea, Cat.No: K-3090) per the manufacturer’s instructions. The extracted total RNA was treated with DNase I to digest the genomic DNA. By using gel electrophoresis and spectrophotometry, the quality and quantity of RNA were determined. After treating the samples with an RNase inhibitor, RNA was converted to cDNA using the PrimeScript RT reagent kit (Takara Bio, Ohtsu, Japan Cat.No. RR037A). Lastly, qRT-PCR analysis was performed on a Rotor-Gene Q instrument (QIAGEN, Germany) using SYBR Green Premix Ex Taq (TaKaRa, Otsu, Shizuoka, Japan, Cat.No: RR420A). The qRT-PCR conditions were 95°C for 10 min (pre‐denaturation) and forty cycles of 95°C for 10 s (denaturation), 61°C for 20 s (annealing), and 72°C for 25 s (extension). As an internal control, beta-actin (β-Actin) was utilized.

### Single-Cell RNA sequencing data processing

Single-cell RNA sequencing data analysis was conducted using Seurat version 4.2.2. The dataset was first loaded and subjected to quality control, removing cells with fewer than 300 or more than 4000 RNA features, or with mitochondrial gene content greater than 10%. Data normalization and scaling were performed using the SCTransform function. Dimensionality reduction and clustering were conducted by selecting the top 15 principal components, followed by clustering with the RunUMAP function. Cell types were annotated using SingleR and previously reported literature. Marker genes for each cell type were identified using the FindAllMarkers function.

### Cell communication analysis

The “CellChat” R package (version 1.5.0) was used to reveal potential intercellular communication mechanisms at the single-cell level. The createCellChat function was employed to construct the CellChat object, and the aggregateNet function was used to describe the signaling emitted from each cell type. Intercellular communication quantities and weights were visualized using the netVisual_circle function, and the netAnalysis_computeCentrality function was used to infer the input and output weights of specific signaling pathways.

### Lactylation scoring

Lactylation-related gene sets were obtained from the GSEA website, comprising a total of 10 pathways. After removing duplicates, 329 lactylation-related genes (LRGs) were identified to form the lactylation gene set. To calculate lactylation scores, we applied the AUCell package, which quantifies the activity of a gene set in individual cells by evaluating whether the expression of genes from the set is enriched in the top-ranking genes of each cell. Specifically, we used the AUCell_calcAUC function to calculate the Area Under the Curve (AUC) for each cell, based on the ranking of gene expression levels. This method allows us to assess the relative expression of the lactylation gene set within each cell, resulting in a lactylation score that reflects the activity of lactylation-related pathways in individual cells.

To ensure robustness, the lactylation scores were calculated for all cells, and we compared these scores across different cell subpopulations to identify those most associated with lactylation activity. The AUCell algorithm’s non-parametric approach makes it ideal for single-cell RNA sequencing data, as it allows for accurate scoring even in cases of sparse gene expression. The resulting lactylation scores were visualized using violin plots generated by ggplot2, providing a clear comparison of lactylation activity across various cell types.

### scMetabolism metabolic analysis

Metabolic characteristics of various cell types within the scRNA-seq data were assessed using the R package scMetabolism. This analysis employed the AUCell scoring principle to evaluate 85 metabolic pathways. Key pathways related to glucose metabolism and lipid metabolism were visualized using bubble plots.

### Pathway and enrichment analysis

Pathway enrichment analysis was conducted using the irGSEA package, a robust R package equipped with multiple scoring functions to assist in pathway scoring. This study integrated AUCell, UCell, and GSVA algorithms, and combined the pathway activation results using the RAA method to calculate the upregulation and downregulation of HALLMARK gene sets. Additionally, the “clusterProfiler” package was utilized for KEGG and GOBP enrichment analyses of plasma cells, with the results visualized using ggplot2.

### Machine learning

To identify genes associated with RA and construct a robust diagnostic model, we employed 12 machine learning algorithms for screening and modeling. These algorithms—Lasso, NaiveBayes, SVM, glmBoost, Enet, plsRglm, XGBoost, LDA, Stepglm, Ridge, RandomForest, and GBM—were selected based on their complementary strengths in handling high-dimensional gene expression data and diverse model requirements. Lasso, Ridge, and Enet are effective for regularization and preventing overfitting, crucial for gene selection. SVM is robust for non-linear classification tasks, while RandomForest and GBM, as ensemble methods, improve predictive accuracy and reduce variance. XGBoost was chosen for its efficiency in handling large datasets with strong predictive performance. Simpler methods like NaiveBayes and LDA were included for their ability to capture probabilistic relationships, while plsRglm and glmBoost provide flexibility with regularization and boosting (34). Stepglm was specifically used for variable selection, identifying the most relevant lactylation-promoting genes. The use of 134 algorithmic combinations ensured comprehensive variable selection and model construction. Diagnostic performance was evaluated using average AUC values from the training and validation sets, ensuring we selected the most predictive and reliable model for RA diagnosis.

### Immune-related analysis

Relative enrichment scores for 29 immune cell types and immune processes were calculated using the GSVA and GSEABase packages, following the ssGSEA strategy. Correlation analyses were performed between immune cell types and the RA lactylation score (RAlac_score). Samples were divided into high RAlac_score and low RAlac_score groups based on the median RAlac_score, and the activation of immune processes was compared between these groups. The expression of immune checkpoint molecules was analyzed for correlation with RAlac_score, and correlation bubble plots were generated using ggplot2.

### Consensus clustering

To further explore the functions of core lactylation genes in RA, unsupervised clustering was performed using the “ConsensusClusterPlus” package, with K=2 selected as the optimal parameter. PCA plots were created using the “FactoMineR” and “factoextra” packages. Differential analysis between the two subgroups was conducted using the Limma package, and heatmaps were generated using the pheatmap package. Enrichment analysis was performed with the “clusterProfiler” package, and visualization was done using the aPEAR package.

### Clinical prediction model construction

To apply the RA lactylation diagnostic model in clinical decision-making, a clinical prediction model was constructed using a multivariate logistics algorithm. The rms package was used for model construction and calibration curve calculation, and ROC curves were plotted using the ROCR package.

## Results

### Integration and quality control of single-cell data

**Figure 1:**
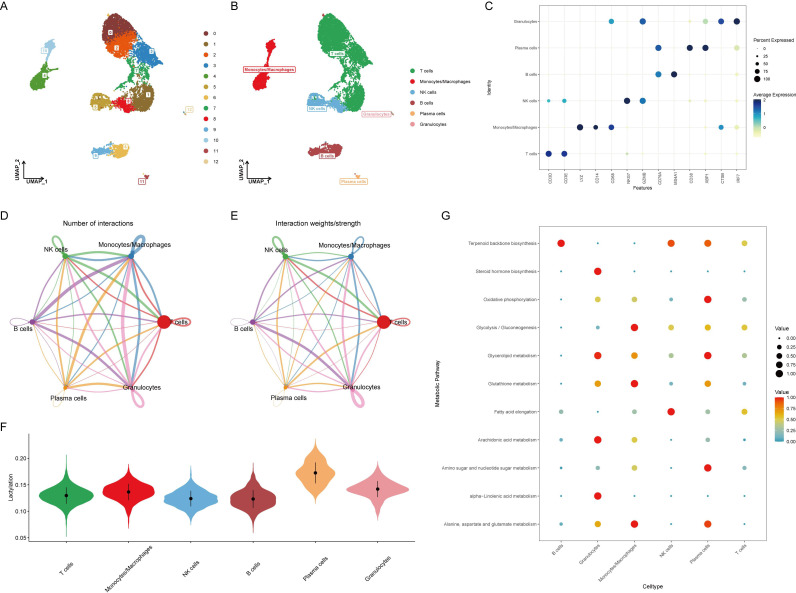
Identification and annotation of cell clusters in RA single-cell data. **(A)** ElbowPlot showing the variance explained by each of the principal components. The top 15 principal components were selected for clustering analysis. **(B)** UMAP plot displaying 13 clusters (0-12) identified from the single-cell RNA sequencing data. Clusters were annotated into six cell types based on SingleR and literature. **(C)** Dot plot showing the expression of marker genes for each annotated cell type: T cells (CD3D, CD3E), monocytes/macrophages (LYZ, CD14, CD68), NK cells (NKG7, GZMB), B cells (CD79A, MS4A1), plasma cells (CD38, XBP1), and granulocytes (CTSB, IRF7). **(D)** Heatmap illustrating the cell-cell communication network, with B cells and plasma cells sending numerous signals to other cell types. **(E)** Bar graph depicting the number of communication signals sent and received by each cell type. **(F)** Violin plot comparing lactylation scores across different cell types, calculated using the AUCell algorithm. Plasma cells show the highest lactylation scores. **(G)** Heatmap showing the expression of key metabolic pathways, including oxidative phosphorylation, glycolysis/gluconeogenesis, and glycerolipid metabolism, across different cell types. Plasma cells exhibit significant upregulation of these pathways.

Before analyzing the lactylation in RA, single-cell data was integrated and quality controlled according to methodological standards, retaining 10,163 cells for downstream analysis. Based on the ElbowPlot results, the top 15 principal components were selected for dimensional reduction clustering, resulting in 13 clusters labeled from 0 to 12 (**Figure 1A**). These clusters were annotated into six cell types according to SingleR and previous literature (**Figure 1B**). The markers for each cell type were as follows: T cells (CD3D, CD3E), monocytes/macrophages (LYZ, CD14, CD68), NK cells (NKG7, GZMB), B cells (CD79A, MS4A1), plasma cells (CD38, XBP1), and granulocytes (CTSB, IRF7) (**Figure 1C**).

### Cellular communication and activation in RA

Evaluation of cell-to-cell communication revealed that B cells and plasma cells sent a large number of signals to other cells, suggesting their activation is related to the development of RA (**Figures 1D, E**). Using the AUCell algorithm, lactylation scores were calculated for each cell type, showing the highest scores in plasma cells (**Figure 1F**). This implies that plasma cells might activate the lactylation process through extensive signaling, altering the surrounding microenvironment and contributing to the onset of RA. Since lactylation involves metabolic processes, the metabolic changes in various cell types were further assessed, with oxidative phosphorylation, glycolysis/gluconeogenesis, and glycerolipid metabolism significantly upregulated in plasma cells, indicating a strong correlation with metabolism (**Figure 1G**).

### Pathway enrichment analysis

**Figure 2:**
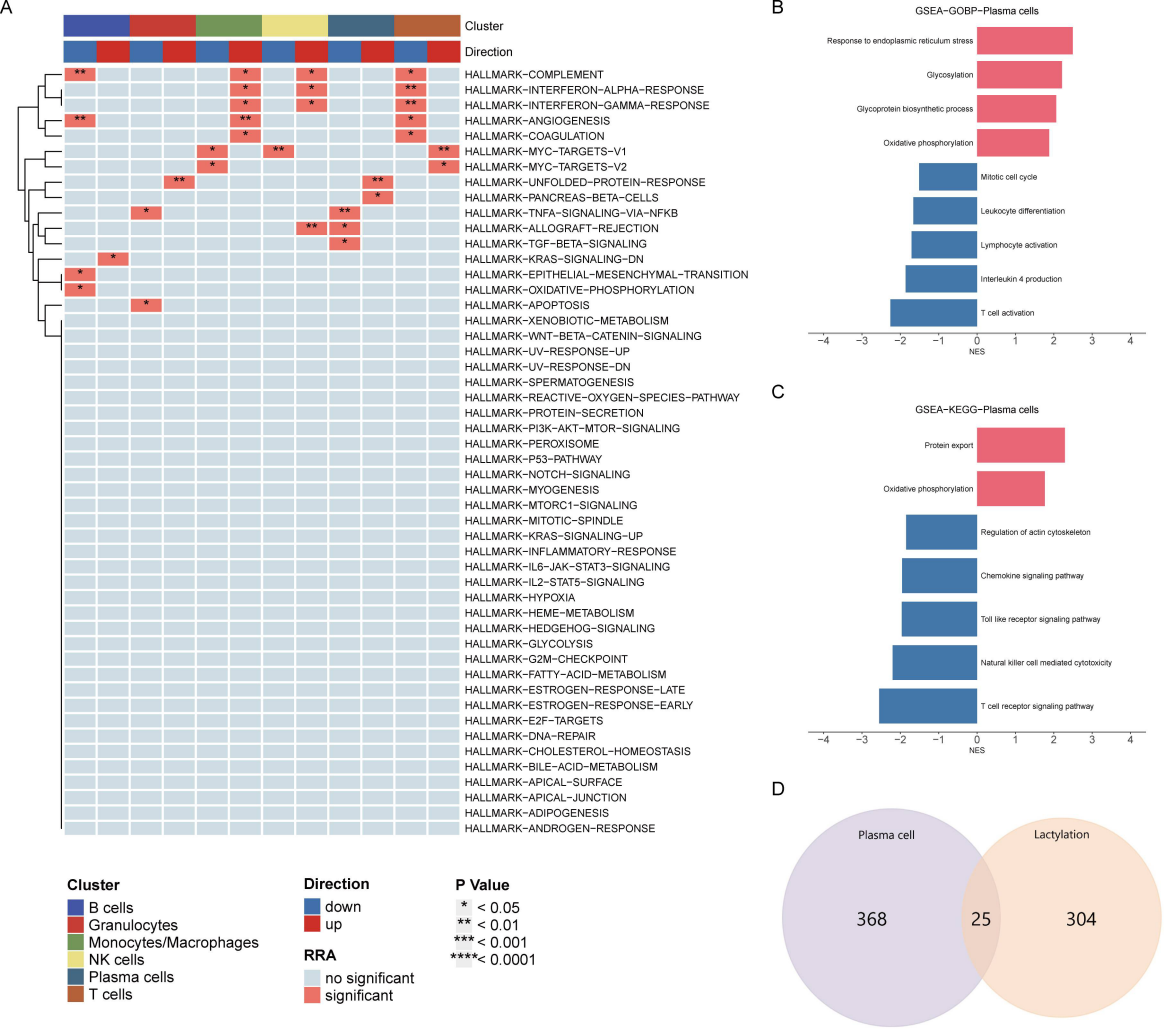
Pathway enrichment analysis of plasma cells. **(A)** HALLMARK pathway enrichment analysis indicating significant activation of the unfolded protein response and downregulation of TNFα and TGFβ pathways in plasma cells. **(B)** GOBP analysis showing upregulation of oxidative phosphorylation, glycosylation, and endoplasmic reticulum stress, with downregulation of immune activation and differentiation in plasma cells. **(C)** KEGG pathway analysis highlighting significant upregulation of oxidative phosphorylation and downregulation of immune chemotaxis in plasma cells. **(D)** Venn diagram displaying the intersection of differentially expressed genes in plasma cells and lactylation-related genes, identifying 25 overlapping genes for further analysis.

HALLMARK pathway enrichment analysis of all cells indicated a significant activation of the unfolded protein response in plasma cells, closely related to endoplasmic reticulum stress, while TNFα and TGFβ pathways were significantly downregulated (**Figure 2A**). Consistent with previous analysis, GOBP analysis showed significant upregulation of oxidative phosphorylation, glycosylation, and endoplasmic reticulum stress, and downregulation of immune activation and differentiation (**Figure 2B**). KEGG analysis similarly revealed significant upregulation of oxidative phosphorylation and downregulation of immune chemotaxis (**Figure 2C**). These results suggest that plasma cells do not accelerate the development of RA through immune activation but rather by activating their metabolic pathways to promote autoantibody production.

### Intersection of Differentially Expressed Genes and Lactylation-Related Genes

Venn diagram analysis showed 25 intersecting genes between highly expressed differential genes in plasma cells and lactylation-related genes. The following analysis will focus on the elucidation of these intersecting genes (**Figure 2D**).

### Identification of Core Lactylation-Promoting Genes in Plasma Cells for RA

**Figure 3:**
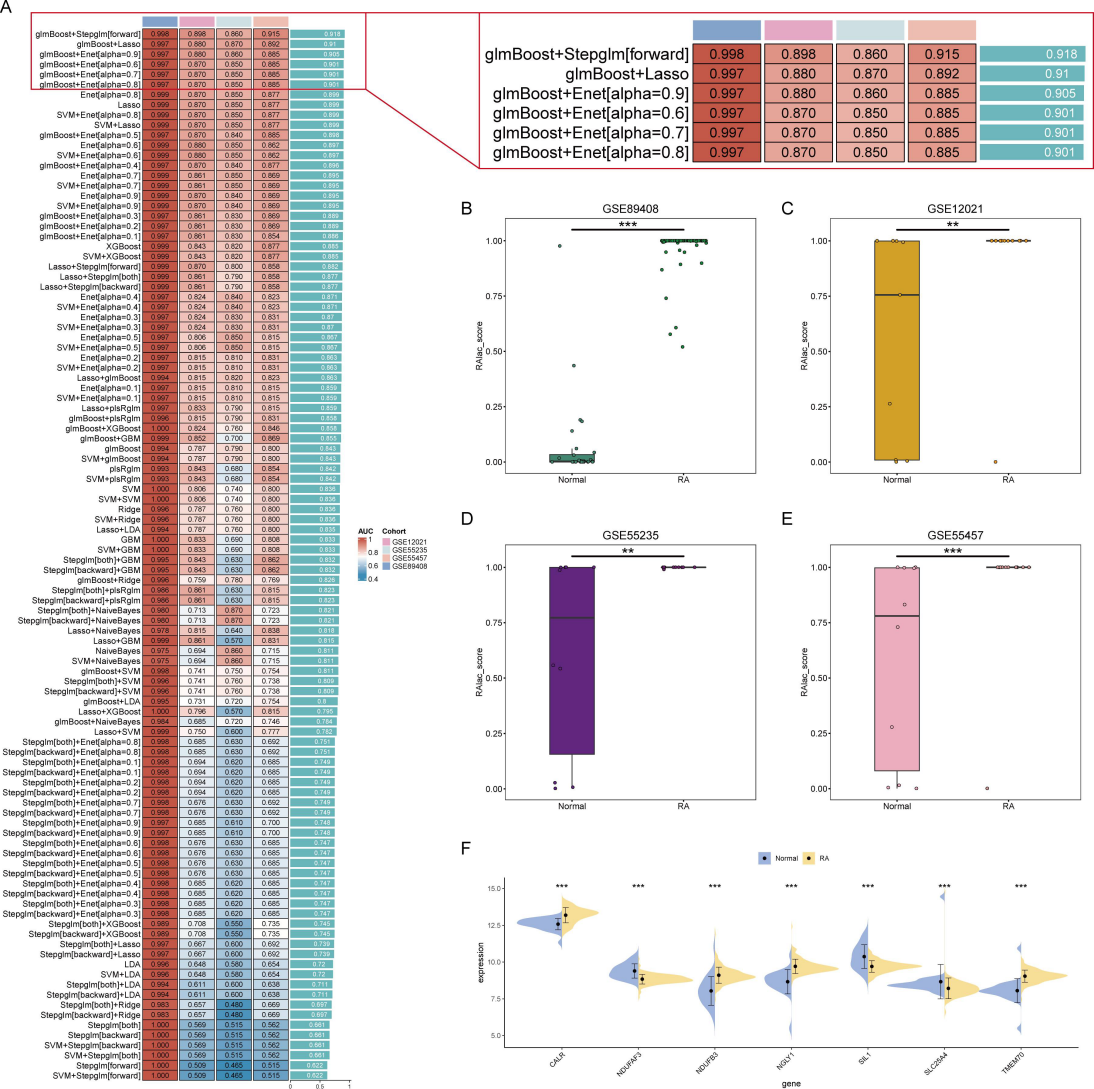
Identification of core lactylation-promoting genes in RA. **(A)** Bar graph showing the average AUC values of various machine learning models. The Stepglm[forward] model, built after gene selection using glmBoost, demonstrates the highest performance with an average AUC of 0.918. **(B-E)** Box plots comparing RAlac_scores between normal and RA groups in the training set and three validation sets. RAlac_scores are significantly higher in the RA group across all datasets. **(F)** Violin plots depicting the expression levels of the seven core lactylation-promoting genes (CALR, NDUFB3, NGLY1, TMEM70, NDUFAF3, SIL1, SLC25A4) in normal and RA samples. CALR, NDUFB3, NGLY1, and TMEM70 are highly expressed in the RA group, while NDUFAF3, SIL1, and SLC25A4 are highly expressed in the control group. ** mean P < 0.01, *** mean P < 0.001.

The role of lactylation in plasma cells is significant in the development of RA. However, the core lactylation-promoting genes remain unidentified. To address this, 134 algorithms across 12 machine learning models were used to screen for key lactylation-promoting genes and construct diagnostic models for RA. Results showed that the Stepglm[forward] model built after gene selection by glmBoost had the highest diagnostic performance, with an average AUC of 0.918 (**Figure 3A**). This diagnostic model retained seven core lactylation genes for RA, allowing the calculation of the RAlac_score for each sample. Both training and validation sets showed significantly higher RAlac_scores in the RA group compared to the normal group, indicating a higher likelihood of developing RA with an elevated RAlac_score (**Figures 3B-E**). Violin plots revealed that CALR, NDUFB3, NGLY1, and TMEM70 were highly expressed in the RA group, while NDUFAF3, SIL1, and SLC25A4 were highly expressed in the control group (**Figure 3F**).

### Correlation between RAlac_score and immune overactivation

**Figure 4:**
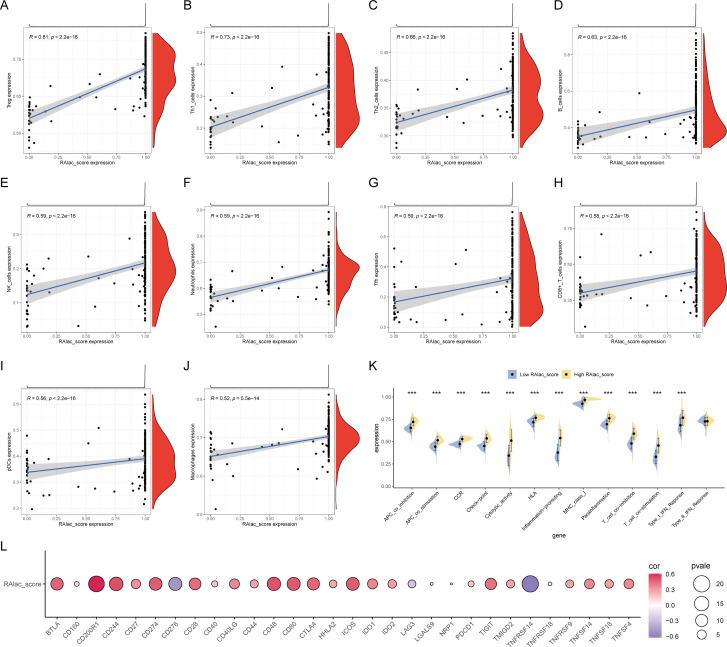
Correlation between RAlac_score and immune activation. **(A-J)** Scatter plots showing significant positive correlations between RAlac_score and various immune cell types, including Treg, Th1, Th2, B cells, NK cells, neutrophils, Tfh cells, CD8+ T cells, pDC, and macrophages. **(K)** Box plots comparing the activation of various immune processes, including immune co-stimulation, between high RAlac_score and low RAlac_score groups. All immune processes are upregulated in the high RAlac_score group. **(L)** Correlation bubble plot illustrating the positive correlation between RAlac_score and the expression of various immune checkpoint molecules, indicating a state of high immune activation in RA patients. ***P w 0.001.

Given the association of RA with immune-inflammatory responses, the relationship between RAlac_score and immune overactivation was evaluated. The results demonstrated a significant positive correlation between RAlac_score and various immune cells, including Treg, Th1, Th2, B cells, NK cells, neutrophils, Tfh cells, CD8+ T cells, pDC, and macrophages, indicating that patients with higher RAlac_scores had more intense immune infiltration and immune responses (**Figures 4A-J**). Similarly, all immune processes, including immune co-stimulation, were upregulated in patients with high RAlac_scores (**Figure 4K**). Most immune checkpoint molecules were positively correlated with RAlac_score, suggesting that RA patients are generally in a state of high immune activation, and immune checkpoint inhibitors (ICI) might be a potential therapeutic approach (**Figure 4L**).

### Diagnostic efficacy of core RA lactylation genes

**Figure 5:**
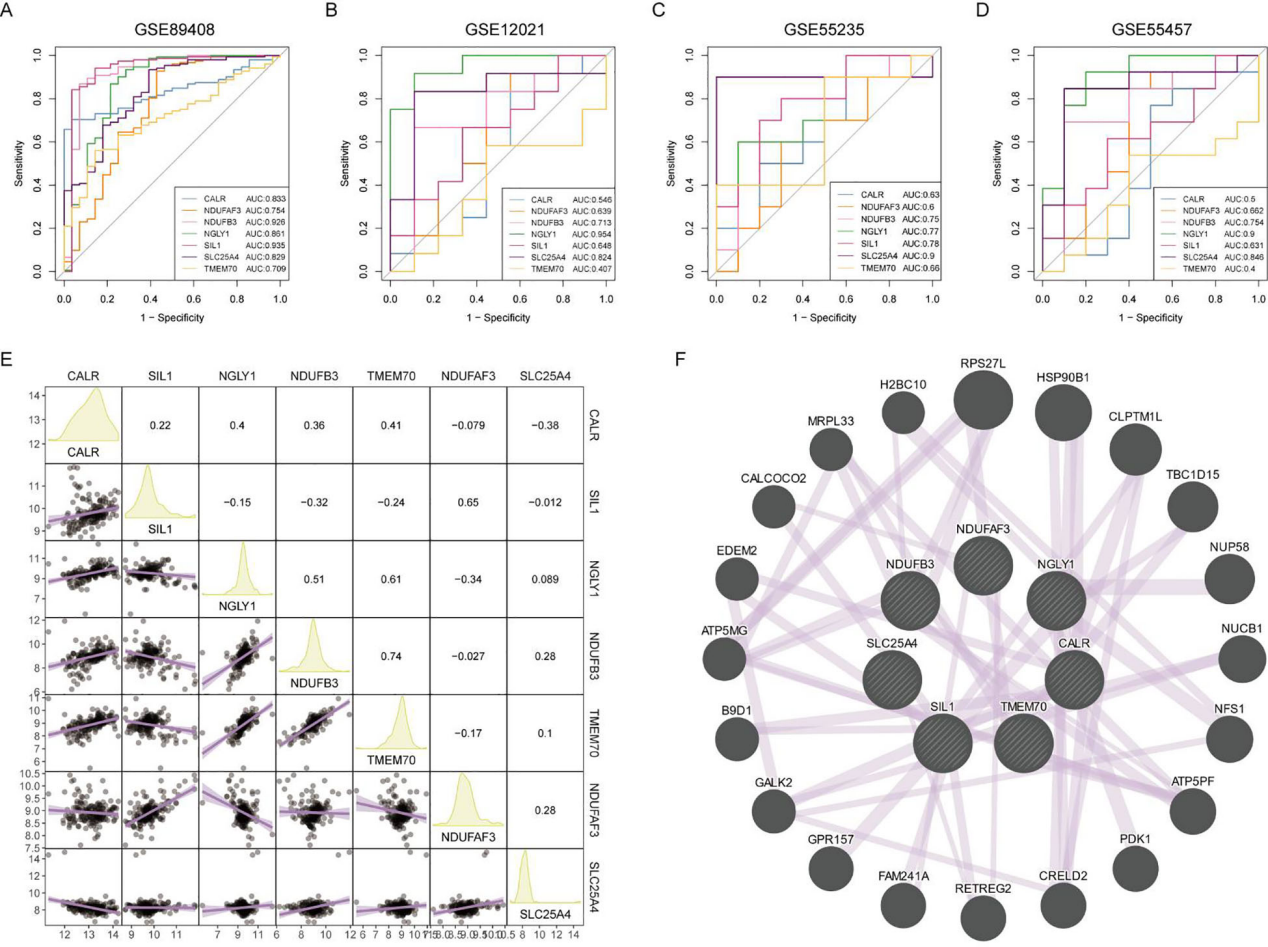
Diagnostic efficacy and expression correlation of core RA lactylation genes. **(A-D)** ROC curves showing the diagnostic efficacy of NDUFB3, NGLY1, SLC25A4, and other core RA lactylation genes, with AUC values ranging from 0.7 to 0.91. **(E)** Correlation heatmap showing the expression relationships between core RA lactylation genes. NGLY1 and CALR, NDUFB3 and CALR, and NDUFB3 and NGLY1 exhibit significant positive correlations, while NDUFB3 and SIL1, and NDUFAF3 and NGLY1 show significant negative correlations. **(F)** GENEMANIA network analysis demonstrating that the proteins expressed by these genes primarily have co-expression relationships.

The diagnostic efficacy of the seven core lactylation genes for RA showed significant differences. Among the four RA gene sets, NDUFB3, NGLY1, and SLC25A4 had the highest diagnostic efficiency, with AUCs ranging from 0.7 to 0.91 (**Figures 5A-D**). The expression correlations between genes also displayed unique characteristics, with significant positive correlations observed between NGLY1 and CALR, NDUFB3 and CALR, and NDUFB3 and NGLY1. In contrast, NDUFB3 and SIL1, and NDUFAF3 and NGLY1 had significant negative correlations (**Figure 5E**). GENEMANIA analysis showed that the proteins expressed by these genes primarily had co-expression relationships (**Figure 5F**).

### Consistent clustering using core RA lactylation genes

**Figure 6:**
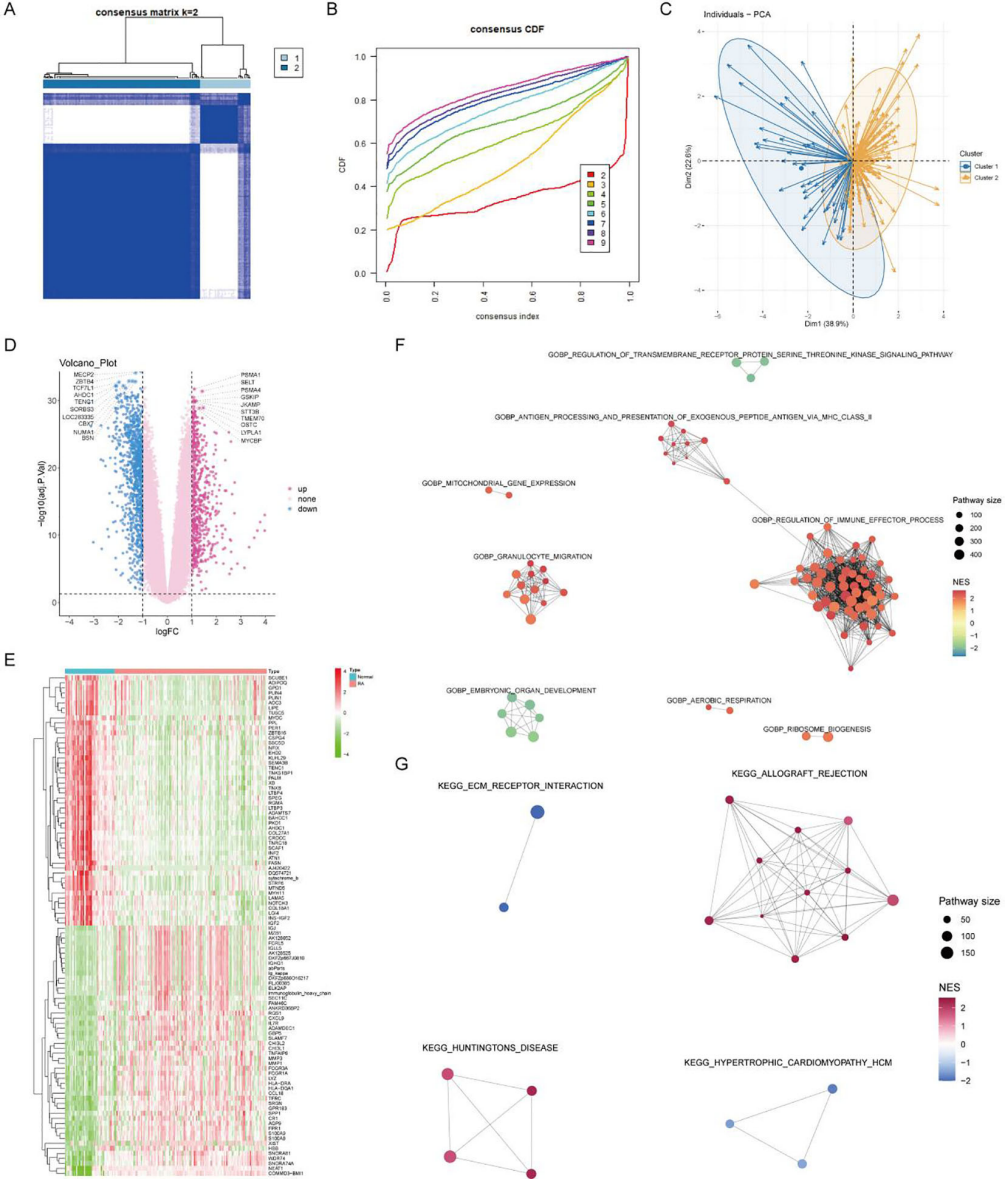
Consensus clustering and differential expression analysis. **(A, B)** CDF plot and clustering heatmap showing stable clustering results with K=2 for the expression matrix of the seven core RA lactylation genes. **(C)** PCA plot demonstrating significant characteristic differences between the two clusters. **(D)** Volcano plot of differential gene expression analysis between the two clusters, with 1621 differentially expressed genes identified (logFC >1 and adjusted P <0.05). **(E)** Heatmap showing the top 50 upregulated and top 50 downregulated genes in the differential expression analysis between the two clusters. **(F, G)** Enrichment analysis plots displaying the GO and KEGG pathways activated in cluster 2, associated with immune activity and extracellular matrix interactions.

Using the expression matrix of the seven core lactylation genes in RA, consistent clustering was performed. Based on the CDF results and the clustering heatmap, k=2 was selected for stable clustering results (**Figures 6A, B**). PCA analysis revealed significant characteristic differences between the two clusters (**Figure 6C**). Differential analysis between the two clusters identified 1,621 differentially expressed genes with logFC absolute value greater than 1 and adjusted P value less than 0.05 (**Figure 6D**). A heatmap displayed the top 50 upregulated and downregulated genes (**Figure 6E**). Enrichment analysis highlighted the GO and KEGG pathways activated in cluster 2, associated with immune activity and extracellular matrix interaction (**Figures 6F, G**).

### Differential expression and pathway activation in clusters

**Figure 7:**
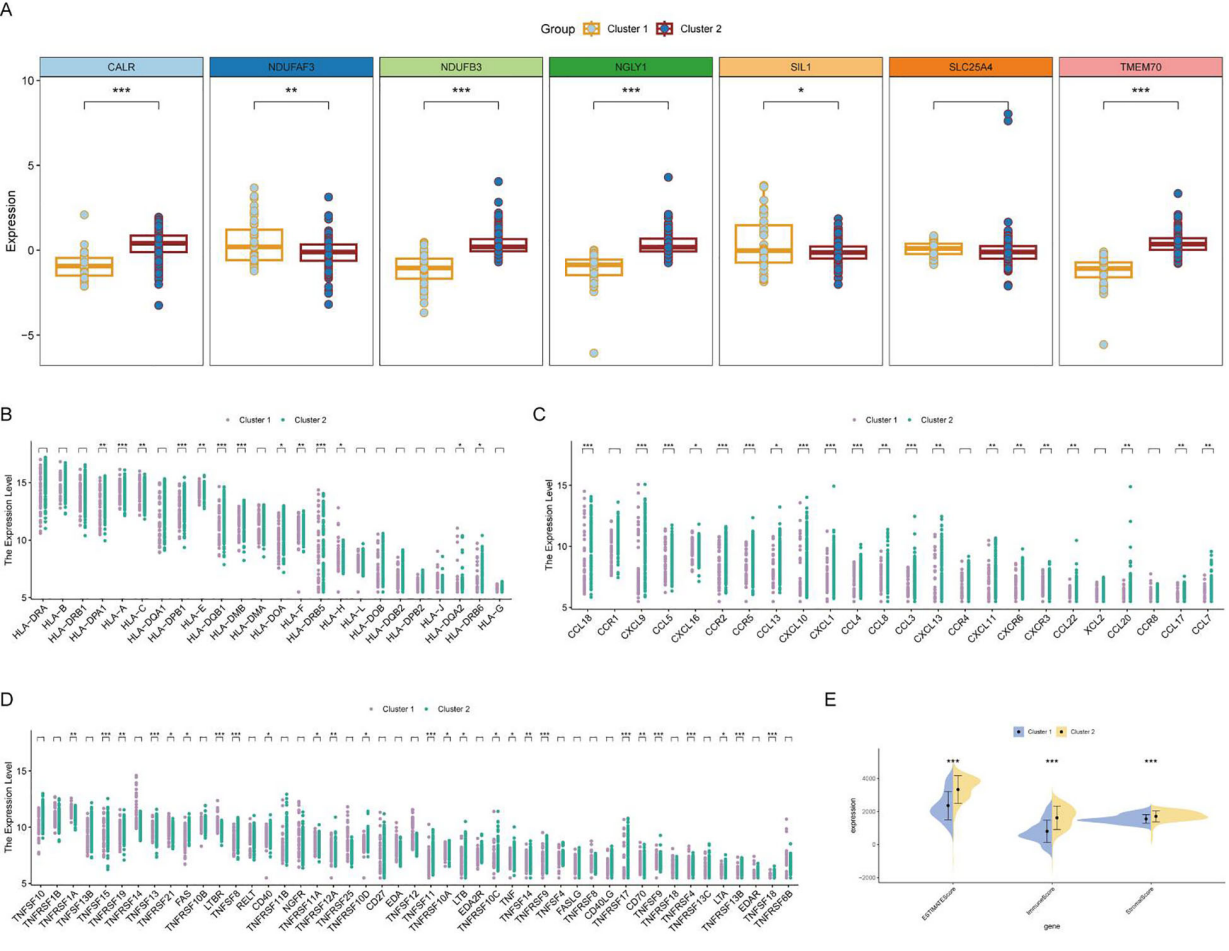
Differential expression and immune activation in RA clusters. **(A)** Box plot showing the differential expression of core lactylation genes between the two clusters. NDUFAF3 and SIL1 are highly expressed in cluster 1, while CALR, NDUFB3, NGLY1, and TMEM70 are highly expressed in cluster 2. **(B)** Heatmap illustrating the expression of MHC molecules. MHC-I molecules (HLA-A, HLA-C) are primarily expressed in cluster 1, whereas MHC-II molecules are highly expressed in cluster 2. **(C, D)** Heatmaps showing the expression of various chemokines and TNF family molecules, which are predominantly expressed in cluster 2, indicating an inflammatory and chemotactic subtype associated with immune activation. **(E)** Box plots comparing the stromal score, immune score, and microenvironment score between the two clusters, calculated using the Estimate algorithm. All scores are significantly higher in cluster 2, suggesting enhanced microenvironment activation. * mean P < 0.05, ** mean P < 0.01, *** mean P < 0.001.

The core lactylation genes showed different expression patterns between the two subgroups, with NDUFAF3 and SIL1 highly expressed in cluster 1, and CALR, NDUFB3, NGLY1, and TMEM70 highly expressed in cluster 2 (**Figure 7A**). Cluster 1 primarily expressed MHC-I molecules such as HLA-A and HLA-C, whereas cluster 2 primarily expressed MHC-II molecules (**Figure 7B**). Most chemokines and TNF family molecules were highly expressed in cluster 2, indicating that cluster 2 is an inflammatory and chemotactic subtype related to immune activation (**Figures 7C, D**). The Estimate algorithm was used to assess the activation of the microenvironment, showing that the stromal score, immune score, and microenvironment score were all significantly higher in cluster 2 (**Figure 7E**).

### Single-cell expression and clinical prediction model

**Figure 8:**
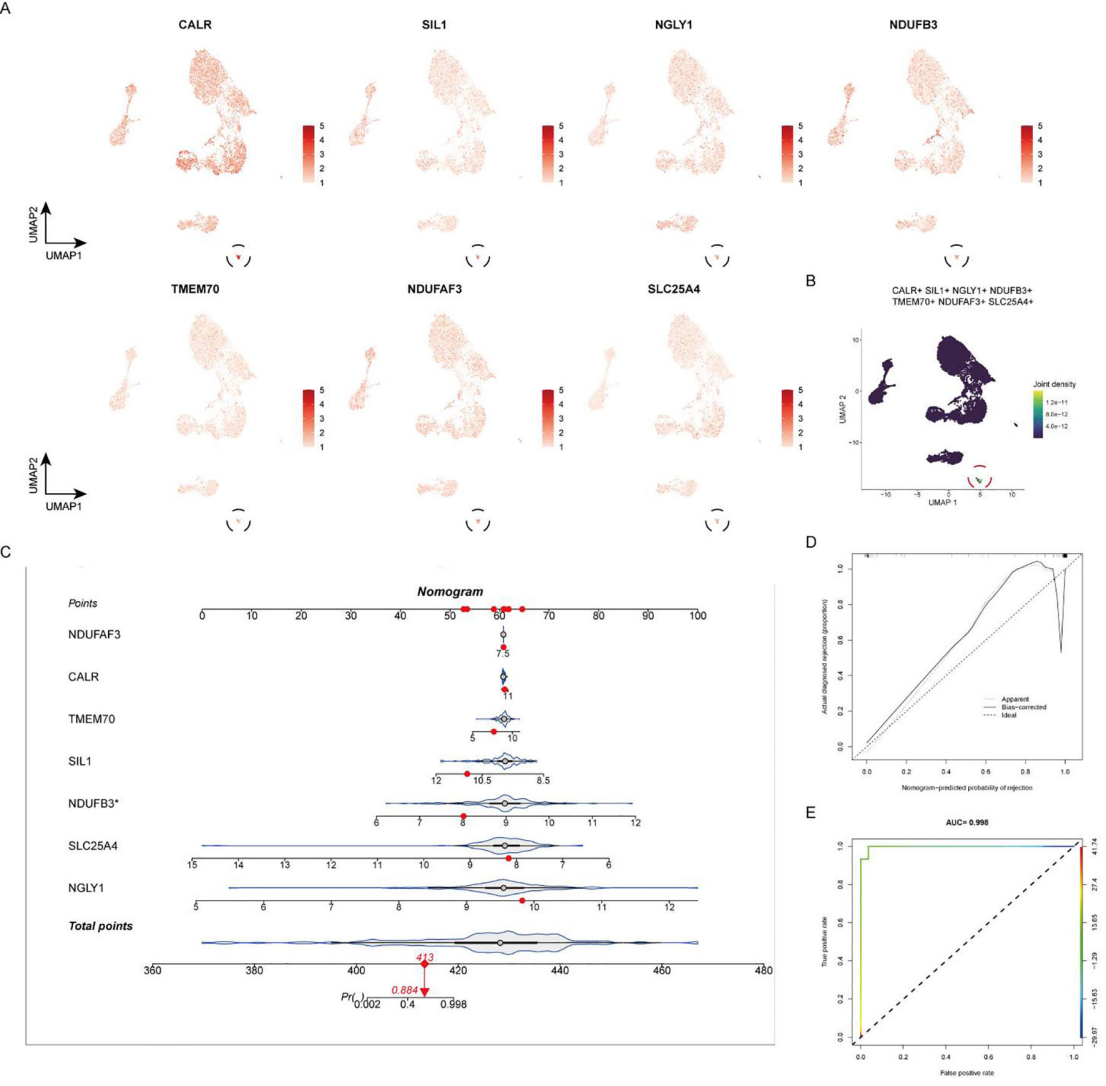
Expression of Core RA Lactylation Genes and Clinical Prediction Model. **(A)** Violin plot depicting the expression of the seven core RA lactylation genes in plasma cells at the single-cell level. **(B)** Density plot showing the aggregated expression of the seven core RA lactylation genes in plasma cells. **(C)** Flowchart illustrating the construction of the clinical prediction model for RA based on core lactylation genes. **(D, E)** ROC curves displaying the performance of the clinical prediction model, with an area under the ROC curve (AUC) exceeding 0.9, indicating good predictive efficacy and calibration.

**Figure 9:**
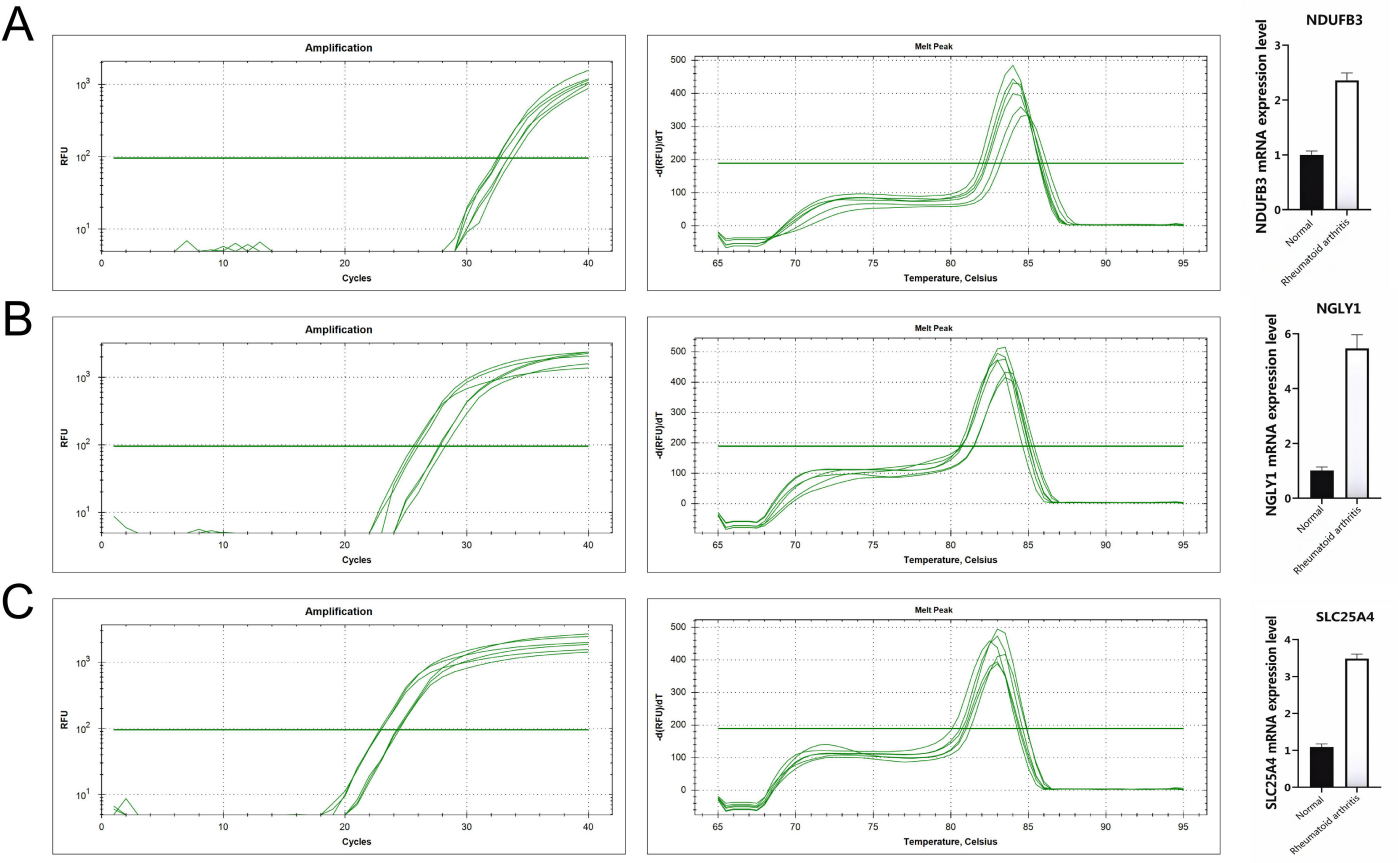
All genes are highly expressed. **(A)** NDUFB3 gene expression. **(B)** NGLY1 gene expression. **(C)** SLC25A4 gene expression.

At the single-cell expression level, all seven RA lactylation genes were distinctly expressed in plasma cells (**Figure 8A**). The aggregated expression density of these seven genes also indicated their presence in plasma cells (**Figure 8B**). Based on the core RA lactylation genes, a clinical prediction model for RA was constructed to evaluate the probability of developing rheumatoid arthritis (**Figure 8C**). This clinical prediction model demonstrated good predictive performance and calibration, with an area under the ROC curve exceeding 0.9 (**Figures 8D, E**). Finally, we found through qRT-PCR experimental analysis that NDUFB3, NGLY1, and SLC25A4 are highly expressed in rheumatoid arthritis compared with normal tissues (**Figure 9**).

## Discussion

The findings of this study highlight the significant role of lactylation in plasma cells in the pathogenesis of RA. By integrating scRNA-seq data and employing advanced bioinformatics and machine learning approaches, we identified core lactylation-promoting genes and constructed a diagnostic model for RA. This discussion will contextualize our findings within the broader landscape of RA research, comparing them with existing studies and elucidating their implications for diagnosis and therapy.

Our study aligns with the growing body of literature that emphasizes the importance of metabolic reprogramming in immune cells during RA. Previous studies have demonstrated that immune cells, particularly T cells and macrophages, undergo metabolic shifts to support their effector functions during inflammation (35). However, the role of plasma cells and their metabolic adaptations in RA has been less explored. Our results add to this emerging understanding by identifying lactylation as a crucial post-translational modification in plasma cells, implicating it in the disease’s metabolic landscape.

Previous research has shown that plasma cells are abundant in the RA synovium and are responsible for the production of autoantibodies such as rheumatoid factor (RF) and ACPAs, which contribute to the disease’s pathogenesis (31, 36). However, the specific metabolic pathways and regulatory mechanisms governing plasma cell function in RA have remained unclear. Our study provides evidence that lactylation is significantly upregulated in plasma cells, suggesting a novel regulatory axis that might influence autoantibody production and immune activation in RA.

The identification of core lactylation-promoting genes and their association with immune activation pathways provides novel insights into the mechanisms underlying RA. Lactylation, as a post-translational modification derived from lactate, has been shown to regulate gene expression and protein function, thus influencing various cellular processes (27). Our findings indicate that plasma cells with high lactylation scores exhibit enhanced metabolic activities, including oxidative phosphorylation and glycolysis, which are essential for sustaining the high energy demands of these cells during antibody production.

Interestingly, our study found a significant positive correlation between the RAlac_score and immune cell infiltration, including Treg, Th1, Th2, B cells, NK cells, and others. This suggests that lactylation in plasma cells might be driving a pro-inflammatory environment in RA. These findings are consistent with recent studies highlighting the role of metabolic reprogramming in supporting the inflammatory functions of immune cells (37). The upregulation of chemokines and TNF family molecules in cluster 2, which had higher lactylation scores, further supports this notion, indicating that these cells are likely contributing to the inflammatory milieu characteristic of RA.

Pathway enrichment analyses revealed that genes upregulated in cluster 2 were associated with immune activity and extracellular matrix interactions, which are critical in the pathogenesis of RA. The involvement of pathways related to oxidative phosphorylation, glycolysis, and endoplasmic reticulum stress in cluster 2 aligns with previous studies that have shown these metabolic processes are crucial for the function and survival of activated immune cells. Moreover, our study identified significant upregulation of MHC-II molecules in cluster 2, which is known to play a role in antigen presentation and T cell activation. This finding suggests that plasma cells with high lactylation scores might be enhancing antigen presentation and subsequent T cell activation, thus perpetuating the inflammatory response in RA. The positive correlation between immune checkpoint molecules and RAlac_score further indicates a state of heightened immune activation, which could be targeted by immune checkpoint inhibitors (ICI) as a potential therapeutic strategy.

The identification of core lactylation-promoting genes and the development of a diagnostic model based on these genes have significant clinical implications. Our diagnostic model, with an area under the ROC curve exceeding 0.9, demonstrates high predictive performance and calibration, suggesting its potential utility in clinical settings for early diagnosis and risk stratification of RA patients. The core lactylation-promoting genes identified in our study, including NDUFB3, NGLY1, SLC25A4, and others, could serve as potential biomarkers for RA. Their expression patterns in plasma cells and their association with metabolic reprogramming and immune activation highlight their relevance in the disease’s pathogenesis. Future studies should validate these findings in larger cohorts and explore their potential as therapeutic targets. Targeting lactylation pathways in plasma cells might modulate their metabolic activities and reduce autoantibody production, thereby mitigating the inflammatory response in RA.

Despite the significant findings, our study has some limitations. The use of scRNA-seq data, while providing high-resolution insights into cellular heterogeneity, is limited by its snapshot nature, capturing gene expression profiles at a single time point. Longitudinal studies are needed to understand the dynamic changes in lactylation and metabolic reprogramming in plasma cells during the progression of RA. Furthermore, while we employed a variety of machine learning algorithms, it is important to acknowledge the limitations associated with these approaches. Different machine learning systems may produce varying results depending on the dataset and parameters used, and the complexity of integrating multiple algorithms can introduce potential biases. Although we aimed to mitigate this by using a comprehensive set of algorithms, further optimization and validation are required to ensure robustness and generalizability. Additionally, functional validation of the diagnostic and therapeutic targets identified by these models is essential. Experimental studies should investigate the specific roles of lactylation-promoting genes in plasma cell function and their contribution to RA pathogenesis. Moreover, the therapeutic potential of targeting lactylation pathways should be explored in preclinical models of RA to assess their efficacy and safety.

In conclusion, our study underscores the significant role of lactylation in plasma cells in the pathogenesis of RA. By integrating scRNA-seq data with advanced bioinformatics and machine learning approaches, we identified core lactylation-promoting genes and developed a highly predictive diagnostic model for RA. These findings provide new insights into the metabolic and immunological mechanisms driving RA and highlight potential biomarkers and therapeutic targets for this debilitating disease. Future directions for research should focus on elucidating the precise molecular mechanisms of lactylation in plasma cells, particularly its role in regulating immune responses and metabolic pathways. Additionally, further experimental studies are needed to validate these findings in larger patient cohorts and explore the therapeutic potential of targeting lactylation pathways in RA treatment.

## Data Availability

The datasets presented in this study can be found in online repositories. The names of the repository/repositories and accession number(s) can be found in the article/supplementary material.
